# Electronic Cigarette Aerosol Is Cytotoxic and Increases ACE2 Expression on Human Airway Epithelial Cells: Implications for SARS-CoV-2 (COVID-19)

**DOI:** 10.3390/jcm10051028

**Published:** 2021-03-03

**Authors:** Kielan Darcy McAlinden, Wenying Lu, Parisa Vahidi Ferdowsi, Stephen Myers, James Markos, Josie Larby, Collin Chia, Heinrich C. Weber, Greg Haug, Mathew Suji Eapen, Sukhwinder Singh Sohal

**Affiliations:** 1 Respiratory Translational Research Group, Department of Laboratory Medicine, School of Health Sciences, College of Health and Medicine, University of Tasmania, Launceston, Tasmania 7248, Australia; kielan.mcalinden@utas.edu.au (K.D.M.); wenying.lu@utas.edu.au (W.L.); Parisa.VahidiFerdowsi@utas.edu.au (P.V.F.); stephen.myers@utas.edu.au (S.M.); jamesmarkos@bigpond.com (J.M.); josie.larby@ths.tas.gov.au (J.L.); collin.chia@ths.tas.gov.au (C.C.); heinrich.weber@ths.tas.gov.au (H.C.W.); greghaug@gmail.com (G.H.); mathew.eapen@utas.edu.au (M.S.E.); 2 Department of Respiratory Medicine, Launceston General Hospital, Launceston, Tasmania 7250, Australia; 3 Department of Pediatrics and Respiratory Medicine, Tasmanian Health Services (THS), North West Hospital, Burnie, Tasmania 7320, Australia

**Keywords:** ACE2 smoking vaping, COVID-19, electronic cigarettes, chronic obstructive pulmonary disease (COPD), SARS-CoV-2

## Abstract

Tobacco smoking has emerged as a risk factor for increasing the susceptibility to infection from severe acute respiratory syndrome coronavirus 2 (SARS-CoV-2) via increased expression of angiotensin-converting enzyme-2 (ACE2) in the lung, linked to coronavirus disease 2019 (COVID-19) development. Given the modifiable nature of electronic cigarettes and the delivery of high concentrations of nicotine, we investigate whether electronic cigarette vaping has the potential to increase susceptibility to SARS-CoV-2 infection. We exposed BEAS-2B cells (bronchial epithelium transformed with Ad12-SV40 2B) and primary small airway epithelial cells (SAECs) to electronic cigarette aerosol condensates produced from propylene glycol/vegetable glycerin or commercially bought e-liquid (±added nicotine) and cigarette smoke extract to investigate if electronic cigarette exposure, like cigarette smoke, increases the expression of ACE2 in lung epithelial cells. In BEAS-2B cells, cytotoxicity (CCK-8), membrane integrity (LDH), and ACE2 protein expression (immunofluorescence) were measured for both 4- and 24 h treatments in BEAS-2B cells and 4 h in SAECs; ACE2 gene expression was measured using quantitative polymerase chain reaction (qPCR) for 4 h treatment in BEAS-2B cells. Nicotine-free condensates and higher concentrations of nicotine-containing condensates were cytotoxic to BEAS-2B cells. Higher LDH release and reduced membrane integrity were seen in BEAS-2B cells treated for 24 h with higher concentrations of nicotine-containing condensates. ACE2 protein expression was observably increased in all treatments compared to cell controls, particularly for 24 h exposures. ACE2 gene expression was significantly increased in cells exposed to the locally bought e-liquid condensate with high nicotine concentration and cigarette smoke extract compared with cell controls. Our study suggests that vaping alone and smoking alone can result in an increase in lung ACE2 expression. Vaping and smoking are avoidable risk factors for COVID-19, which, if avoided, could help reduce the number of COVID-19 cases and the severity of the disease. This is the first study to utilize electronic cigarette aerosol condensates, novel and developed in our laboratory, for investigating ACE2 expression in human airway epithelial cells.

## 1. Introduction

A novel coronavirus emerged at the end of 2019, the spread of severe acute respiratory syndrome coronavirus 2 (SARS-CoV-2) has since consumed the planet, with the cruel development of coronavirus disease 2019 (COVID-19) in some [1,2]. This virus has resulted in one of the deadliest human pandemics in modern history [3]. As of the 26th of February 2021, 112,649,371 confirmed cases of COVID-19 and 2,501,229 confirmed deaths had been recorded worldwide [4]. The deadliness of the novel coronavirus is heightened by the remarkably strong binding affinity between the virus and human airway cells [5,6]. The binding affinity for SARS-CoV-2 is far higher than previously detected human strains of coronavirus. Early basic reproduction number, R0 value estimates for SARS-CoV-2 also range from 2.2 to as high as 6.47 [7,8,9]. Sadly, this pandemic is culminated by the perfect storm of a highly contractive and strongly transmissible nature.

The glycosylated spike proteins on the surface of the SARS-CoV-2 virus mediate host cell entry via the binding with angiotensin-converting enzyme-2 (ACE2) on human epithelial cells of the upper and lower respiratory tract [10,11]. ACE2, known for its role in vasodilation [12], is also implicated as the high-affinity binding site for SARS-CoV-2 [13]. Smokers’ increased susceptibility to the SARS-CoV-2 virus, the risk for severe disease progression, and large representation in COVID-19 morbidity and mortality are increasingly documented [14,15,16,17,18]. Previous studies have also exposed the link between tobacco cigarette smoking and the increased expression of ACE2 in various epithelial cells of the smoker’s lungs [19,20,21]. We and others have shown in human tissue (smokers, chronic obstructive pulmonary disease, COPD) that the expression of ACE2 was increased in type-2 pneumocytes, alveolar macrophages, and small airway epithelium in comparison with healthy control tissue [22,23,24,25]. Similar changes have been observed for increased epithelial ACE2 expression in large airway endobronchial biopsies from patients with COPD compared to asthmatics [26]. The link has since been made with nicotinic receptors, indicating that nicotine induces ACE2 overexpression through mediating α7-subtype nicotinic receptors (α7-nAChR) [27]. Lee et al. have further demonstrated that smoking or vaping may critically exacerbate COVID-19-related inflammation as well or increase susceptibility to COVID-19 through ACE2 [28]. Gaiha and colleagues suggested the need for education and training as they found youth use of electronic cigarettes is associated with COVID-19 [29].

We believe that, along with traditional tobacco smoking and heat-not-burn devices, e-cigarette vaping can increase the expression of ACE2 in the lungs and, in turn, raise the individual’s susceptibility to SARS-CoV-2 infection and subsequent development of COVID-19 [30]. This comes on top of a multitude of other observed pathophysiological changes in respiratory cells following exposure to vape [31]. Here we generate e-cigarette aerosol condensates and aim to measure cytotoxic effects along with the mRNA and protein expression levels of ACE2 following exposure to immortalized human bronchial epithelial cells (BEAS-2B). We have also included initial findings in primary human small airway epithelial cells (SAECs) in which we measured ACE2 protein expression following 4 h treatment.

## 2. Materials and Methods

### 2.1. Electronic Cigarette Aerosol Condensate Production

**Figure 1:**
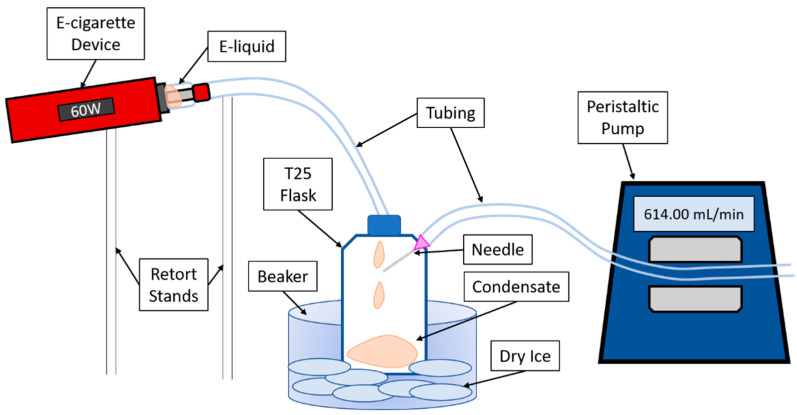
Diagram of electronic cigarette aerosol condensate generation.

Drag2 device (Voopoo) with 0.4Ω U2 dual coil (Voopoo) was used for the generation of e-cigarette aerosol, operated at 60 watts. Watermelon and menthol (WM)-flavored e-liquid (Juicius Maximus, VapeTrail) was locally acquired commercially available (Launceston, Tasmania, Australia) and tested alongside propylene glycol/vegetable glycerin (PG/VG, 50%/50%) alone and cigarette smoke extract (CSE) (1R6F, Kentucky Tobacco Research & Development Center, Lexington, KY, USA). CSE was extracted in 1mL of dimethyl sulfoxide (DMSO) (100%, D2650, Sigma, North Ryde BC, New South Wales, Australia). Nicotine (N3876-100, Sigma, North Ryde BC, New South Wales, Australia) was added to e-liquids at concentrations of both 18 mg/mL and 60 mg/mL. For every e-liquid condensate with and without nicotine, a separate coil and apparatus were used, and coils were not allowed to overheat. Condensates were collected in a T25 flask on a bed of dry ice with the aid of a vacuum pump set to 614 mL/min (Masterflex, John Morris, Melbourne, Victoria, Australia). This procedure of condensate collection is novel and was developed in our laboratory, details are depicted in Figure 1. Collected condensate was diluted in media to working concentrations (1–0.05%) for all experiments.

### 2.2. Cell Culture

BEAS-2B cells were purchased from American Type Culture Collection (ATCC^®^ CRL-9609^TM^, In Vitro Technologies, Melbourne, Victoria, Australia) were cultured in BEGM^TM^ bronchial epithelial cell growth medium BulletKit^TM^ (CC3170, Lonza, Mount Waverley, Victoria, Australia) containing BEGM^TM^ basal medium (CC-3171, Lonza, Mount Waverley, Victoria, Australia) and BEGM^TM^ SingleQuots^TM^ supplements (CC-4175, Lonza, Mount Waverley, Victoria, Australia) supplemented with 1% penicillin/streptomycin (P4333, Sigma, North Ryde BC, New South Wales, Australia) and 1% amphotericin B (A2942, Sigma, North Ryde BC, New South Wales, Australia). Human small airway epithelial cells (SAECs) (CC-2547, purchased from Lonza, Mount Waverley, Victoria, Australia) were cultured in SAGM^TM^ small airway epithelial cell growth medium BulletKit (CC-3118, Lonza, Mount Waverley, Victoria, Australia) containing SABM^TM^ basal medium (CC-3119, Lonza, Mount Waverley, Victoria, Australia) and SAGM SingleQuots^TM^ supplements (CC-4124, Lonza, Mount Waverley, Victoria, Australia) supplemented with 1% penicillin/streptomycin (P4333, Sigma, North Ryde BC, New South Wales, Australia) and 1% amphotericin B (A2942, Sigma, North Ryde BC, Australia), grown in flasks coated in bovine collagen I (A10644-01, Life Technologies, Scoresby, Victoria, Australia). Cells were maintained in a humidified incubator (37 °C, 5% CO_2_).

### 2.3. Cytotoxicity and Viability Measurements

BEAS-2B cells were seeded at a density of 2.5 × 10^4^ 24 h prior to treatment with increasing concentrations of CSE (0.5%, 1%) and e-cigarette aerosol condensate (0.05%, 0.1%, 0.5%, 1%) in freshly applied media for 4 h and 24 h, and cell viability (cell counting kit-8 (CCK-8), (96992, Sigma, North Ryde BC, New South Wales, Australia) and lactate dehydrogenase (LDH) (G1780, Promega Corporation, Alexandria, New South Wales, Australia) assays were performed as per the manufacturer’s guidelines. 100% DMSO treatment just before the addition of CCK-8 reagent was used as a positive control for total killing” in the CCK-8 assay. While for the LDH assay, 10 μL of 10× lysis solution was added for maximal LDH release. For viability, absorbance at 450 nm was recorded 1.5 h following the addition of CCK-8 reagent. LDH activity was measured in supernatants by recording the absorbance at 490 nm immediately after the addition of the stop solution using a plate reader (Infinite M200 Pro, Tecan Group Ltd., Mannedorf, Switzerland). CCK-8 data are presented as viability (%) in comparison with cell controls, while LDH data are represented as a percentage of maximal LDH release. Experiments were replicated at least three times for both assays with treatments in triplicate or quadruplicate.

### 2.4. qPCR

BEAS-2B cells were seeded at a density of 6.25 × 10^4^ for 24 well plates 24 h prior to treatment with concentrations of CSE (0.5%, 1%) and e-cigarette aerosol condensate (0.1%) in freshly applied media for 4 h before cells were lysed and total RNA was isolated using the ISOLATE II RNA mini kit (BIO52072, Bioline, Eveleigh, New South Wales, Australia). cDNA was prepared from 1 µg of RNA template using the high-capacity cDNA reverse transcription kit (4368814, Applied Biosystems, Life Technologies, Scoresby, Victoria, Australia) according to the manufacturer’s instructions. Quantitative real-time PCR was performed by applying the StepOnePlus™ real-time PCR system with TaqMan™ Fast Advanced Master Mix and FAM-labeled primers (4444557, Applied Biosystems, Life Technologies, Scoresby, Victoria, Australia), ACE2 (Hs01085333_m1), and β-actin (ACTB) (Hs01060665_g1), according to the manufacturer’s recommendations. All the samples were run in duplicate and normalized to ACTB. The average cycle threshold (Ct) value of the three independent repeats was used for the comparative Ct (ΔΔCt) method, and the relative gene expression was done using the 2^−ΔΔCt^ method.

### 2.5. Immunofluorescence

Cells were seeded into 8-well chambers (Millicell EZSLIDE 8-well, Merck, North Ryde BC, New South Wales, Australia) at a density of 2 × 10^4^, allowed to reach 70–80% confluence in 24 h, before treating with condensates (0.1%) or CSE (0.5%, 1%) for either 4 h or 24 h. SAECs were seeded into 8-well chambers coated with bovine collagen I. Cells were then fixed and permeabilized in 4% paraformaldehyde/0.1% Triton X-100, blocked with 10% goat serum for 45 min and incubated with primary ACE2 monoclonal antibody (1:100; ab89111, AbCam, Melbourne, Victoria, Australia) overnight at 4 °C. In each experiment, a negative control was achieved through the replacement of the primary antibody with mouse IgG1 (X0931, Dako, Mulgrave, Victoria, Australia). Goat anti-mouse secondary antibody (1:500; Alexa Fluor 488, Invitrogen, Scoresby, Victoria, Australia) was applied for an hour at room temperature before staining nuclei with DAPI (Invitrogen, Scoresby, Victoria, Australia) for a minute and mounted with Fluoromount mounting medium (Invitrogen, Scoresby, Victoria, Australia).

### 2.6. Imaging

Immunofluorescence was imaged using the Olympus FV1200 confocal laser scanning microscope (Olympus Life Science Europe GmbH, Hamburg, Germany). Alexa Fluor, 488 images were captured under excitation: 470–495 nm, dichroic beam splitter 505 nm, and emission of 510–650 nm. The images were acquired at 40× objectives and taken at a speed of 2 µs/pixel. Brightfield images of plated cells were obtained via a digital inverted microscope, acquired at 40× (EVOS, Invitrogen, Scoresby, Victoria, Australia).

### 2.7. Statistics

GraphPad (La Jolla, CA, USA) version 8.0 was used for statistical analysis using Kruskal–Wallis test in combination with Dunn’s multiple comparison test or Mann-Whitney U test. Significance was deemed to be *p* < 0.05.

## 3. Results

### 3.1. Exposure to Electronic Cigarette Aerosol Condensate and Cigarette Smoke Is Toxic to Bronchial Epithelial Cells (BEAS-2B)

**Figure 2:**
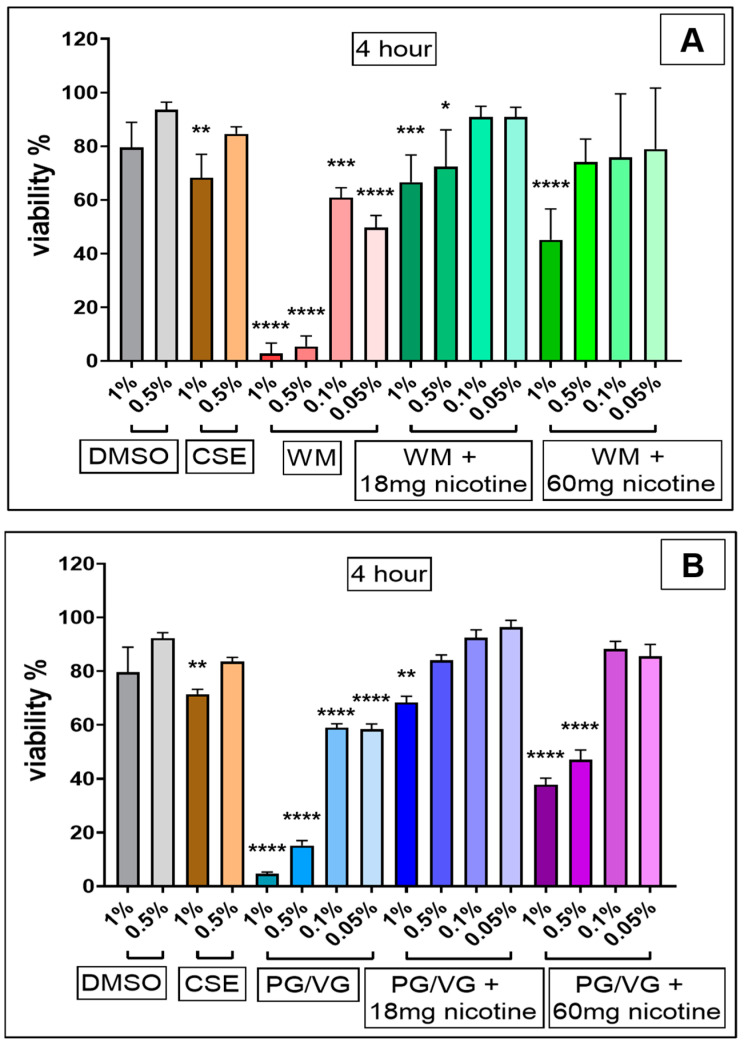
Viability of Bronchial epithelial (BEAS-2B) cells following 4 h electronic cigarette aerosol and cigarette smoke exposure: BEAS-2B cells were exposed to increasing concentrations (0.05%, 0.1%, 0.5% and 1%) of electronic cigarette condensate or cigarette smoke extract (CSE, 1% and 0.5%) for 4 h. Electronic cigarette condensates were generated from (**A**) watermelon (WM)-flavored e-liquid (Juicius Maximus) ± nicotine (18 mg/mL and 60 mg/mL); and (**B**) propylene glycol/vegetable glycerin (PG/VG) ± nicotine (18 mg/mL and 60 mg/mL). Viability was measured in cells treated for 4 h using a cell counting kit-8 (CCK-8) assay and is presented as percent viability in comparison with cell control. Data are displayed as median with interquartile range of *n* = 3–4 independent experiments. Kruskal–Wallis test with Dunn’s multiple comparison test was performed. * *p* < 0.05; ** *p* < 0.01; *** *p* < 0.001; **** *p* < 0.0001. DMSO, dimethyl sulfoxide; CSE, cigarette smoke extract.

**Figure 3:**
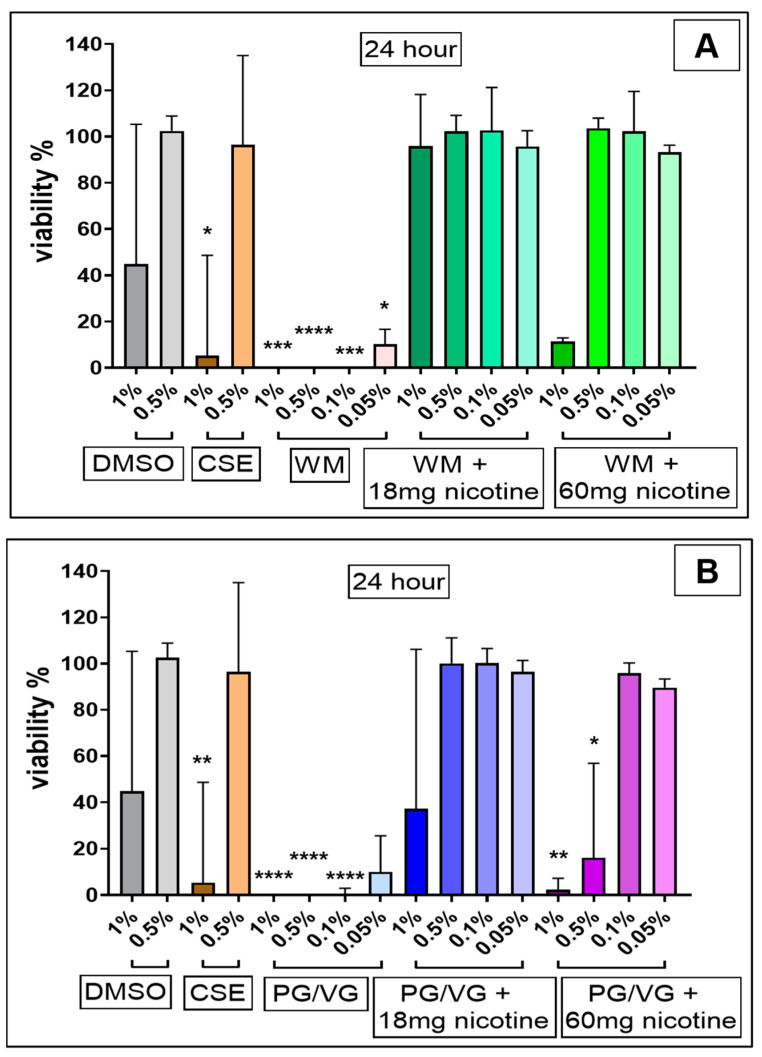
Viability of BEAS-2B cells following 24 h electronic cigarette aerosol and cigarette smoke exposure: Bronchial epithelial (BEAS-2B) cells were exposed to increasing concentrations (0.05%, 0.1%, 0.5% and 1%) of electronic cigarette condensate or cigarette smoke extract (CSE, 1% and 0.5%) for 24 h. Electronic cigarette condensates were generated from (**A**) watermelon (WM)-flavored e-liquid (Juicius Maximus) ± nicotine (18 mg/mL and 60 mg/mL); and (**B**) propylene glycol/vegetable glycerin (PG/VG) ± nicotine (18 mg/mL and 60 mg/mL). Viability was measured in cells treated for 24 h using a CCK-8 assay and is presented as percent viability in comparison with cell control. Data are displayed as median with interquartile range of *n* = 3–4 independent experiments. Kruskal–Wallis test with Dunn’s multiple comparison test was performed. * *p* < 0.05; ** *p* < 0.01; *** *p* < 0.001; **** *p* < 0.0001.

CSE (1%) treatment significantly reduced viability at both 4 h (Figure 2; *p* < 0.01) and 24 h (Figure 3; *p* < 0.05) in comparison with cell controls and independent of the original DMSO solvent. 24 h post-treatment with nicotine-free WM e-liquid and PG/VG condensates (Figure 3) resulted in total cell death at 1%, 0.5%, and 0.1% (*p* < 0.0001, *p* < 0.001) concentrations tested, with significant decrease also observed in low concentrations of 0.05% for WM (*p* < 0.05) (Figure 3A). Such reduction in viability was also observed for higher concentrations (1%, 0.5%) of condensates without nicotine following 4 h treatment (*p* < 0.0001), which faded in lower concentration (0.1%, 0.05%) (*p* < 0.0001, *p* < 0.001) (Figure 2). Interestingly in comparison to non-nicotine bases, condensates with nicotine content of 18 mg/mL and 60 mg/mL in WM-flavored e-liquid and PG/VG showed greater viability at 0.1% and 0.05% concentration at 4 h (Figure 2) and 24 h (Figure 3) post-treatment. 4 h exposures to 1% and 0.5% concentrations of these condensates (WM or PG/VG with nicotine) did however result in significant reduction in viability (*p* < 0.0001; *p* < 0.001; *p* < 0.05) (Figure 2). This significantly reduced viability was also noticed 24 h post-exposure to similar higher concentrations (1%, 0.5%) of condensates from PG/VG with 60 mg/mL nicotine (Figure 3B; *p* < 0.01; *p* < 0.05) and high concentration (1%) condensate from WM with 60 mg/mL nicotine (Figure 3A; ns). The percentage of cytotoxicity, as determined by CCK-8 assay, increases over time (from 4 h to 24 h; Figure 6). Comparatively, the degree of change in %viability between 4 and 24 h treatments is higher in nicotine containing condensates and CSE, with greatest change seen in WM and PG/VG with 60 mg/mL nicotine, and CSE (Figure 6).

### 3.2. Cellular Membrane Damage from e-Cigarette Aerosol Condensate and Cigarette Smoke Extract Are Distinct Following 24 h Exposure

**Figure 4:**
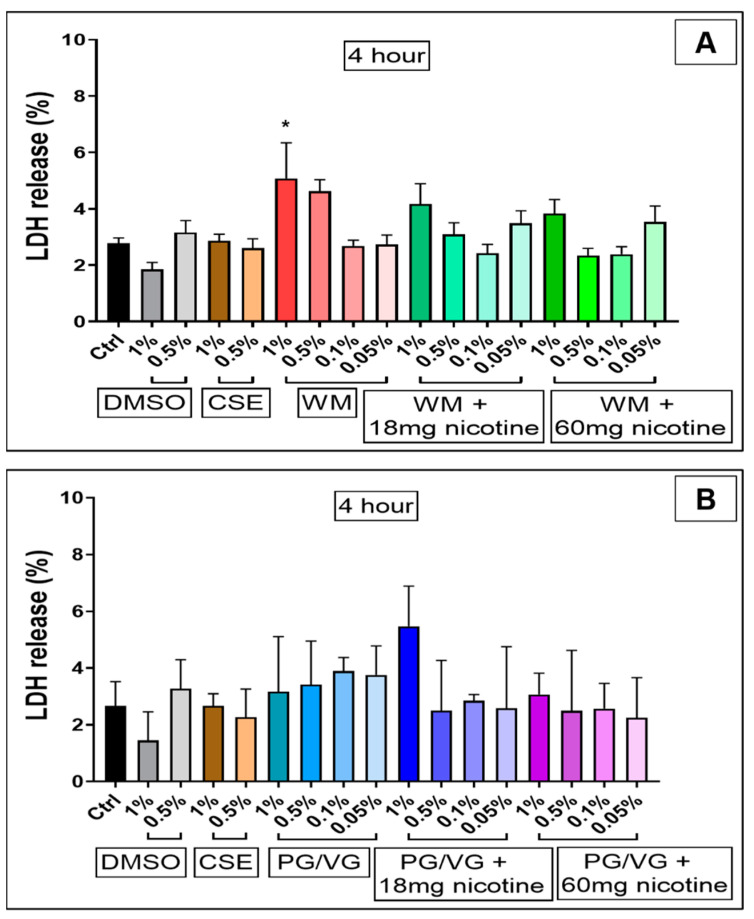
Membrane integrity of BEAS-2B cells following 4 h electronic cigarette aerosol and cigarette smoke exposure: Bronchial epithelial (BEAS-2B) cells were exposed to increasing concentrations (0.05%, 0.1%, 0.5% and 1%) of electronic cigarette condensate from (**A**) watermelon (WM) e-liquid) or (**B**) propylene glycol/vegetable glycerin (PG/VG) ± nicotine or cigarette smoke extract (CSE, 1% & 0.5%) for 4 h. Lactate dehydrogenase (LDH) release was measured from cells treated for 4 h and is presented as the percentage of maximal LDH release. Data are displayed as median with an interquartile range of *n* = 3–4 independent experiments. Kruskal–Wallis test with Dunn’s multiple comparison test was performed. * *p* < 0.05.

**Figure 5:**
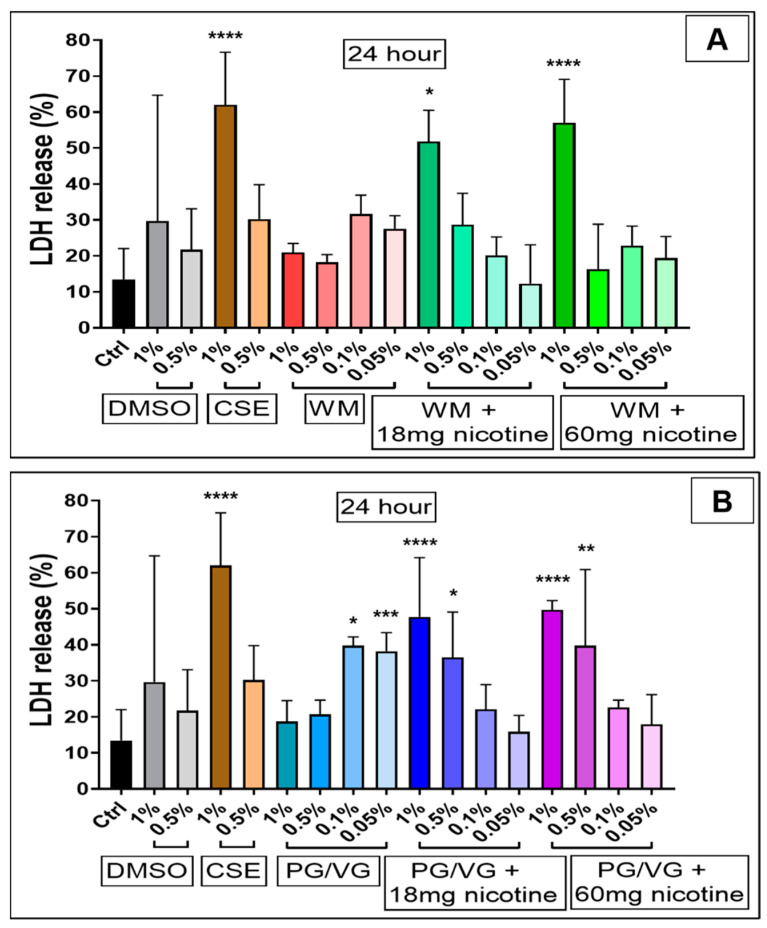
Membrane integrity of BEAS-2B cells following 24 h electronic cigarette aerosol and cigarette smoke exposure: Bronchial epithelial (BEAS-2B) cells were exposed to increasing concentrations (0.05%, 0.1%, 0.5% and 1%) of electronic cigarette condensate from (**A**) watermelon (WM) e-liquid) or (**B**) propylene glycol/vegetable glycerin (PG/VG) ± nicotine or cigarette smoke extract (CSE, 1% and 0.5%) for 24 h. Lactate dehydrogenase (LDH) release was measured from cells treated for 24 h and is presented as the percentage of maximal LDH release. Data are displayed as median with an interquartile range of *n* = 3–4 independent experiments. Kruskal–Wallis test with Dunn’s multiple comparison test was performed. * *p* < 0.05; ** *p* < 0.01; *** *p* < 0.001; **** *p* < 0.0001.

**Figure 6:**
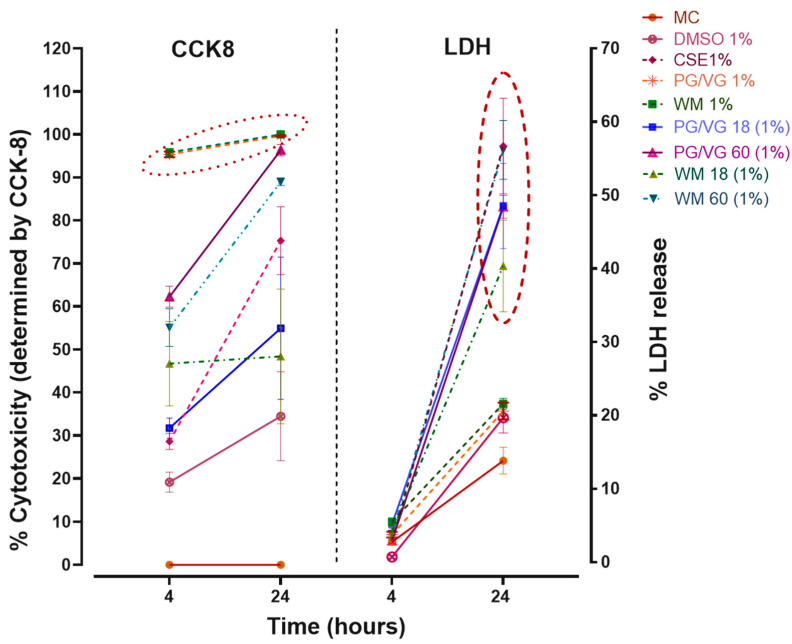
Time- and nicotine-dependent changes in LDH release and cytotoxicity of BEAS-2B cells following electronic cigarette aerosol and cigarette smoke exposure: Graphical representation comparing percent cytotoxicity (**left**) and percent lactate dehydrogenase (LDH) release (**right**) in BEAS-2B cells for 4 and 24 h post-treatment with condensates (1%) with and without nicotine, and cigarette smoke extract (CSE1%) and DMSO1%, with non-treated cells as controls (MC). Overall percentage increase in cytotoxicity was noticed in all treatments over controls. A nicotine-associated differential effect was accentuated in CCK-8 results for most nicotine-containing condensates (PG/VG 18 mg, PG/VG 60 mg, WM 60 mg, CSE) but is absent for PG/VG 1% and WM 1% (marked in a dotted circle). Watermelon-flavored e-liquid and PG/VG nicotine-containing condensates (marked in the red dashed circle) showed a higher percent LDH release similar to CSE treatment, while watermelon (WM) and PG/VG on their own showed lower LDH release. *n* = 3–4 independent experiments.

LDH release 4 h post-treatment (Figure 4) was lower than 24 h, with cellular disruption likely yet to occur; however, we observe a slight relative increase in LDH release for 1% WM (*p* < 0.05; Figure 4A). Treatment with CSE (1%) (*p* < 0.0001) and high concentrations (1%) of e-cigarette condensates containing nicotine (18 mg/mL and 60 mg/mL) (*p* < 0.0001; *p* < 0.05) for 24 h resulted in large increases in LDH release (Figure 5), thus indicating cell membrane disruption and damage in these cells (Figure 5A,B). Percentage LDH release from 24 h condensate treatment (1% with nicotine) was comparable to CSE treatments at a similar dose. Exposure to high concentrations (0.5%) of condensates from PG/VG with 18 mg/mL and 60 mg/mL nicotine (*p* < 0.05; *p* < 0.01) also significantly increased LDH release post 24 h exposure (Figure 5B). Increasing levels of LDH release are noticeably pronounced after 24 h, and a nicotine-dependent accentuation in cellular membrane disruption in BEAS-2B cells was observed (Figure 6).

### 3.3. Notable Morphological Changes to BEAS-2B Cells Resulting from 24 h Exposure from e-Cigarette Aerosol Condensate and Cigarette Smoke Extract

**Figure 7:**
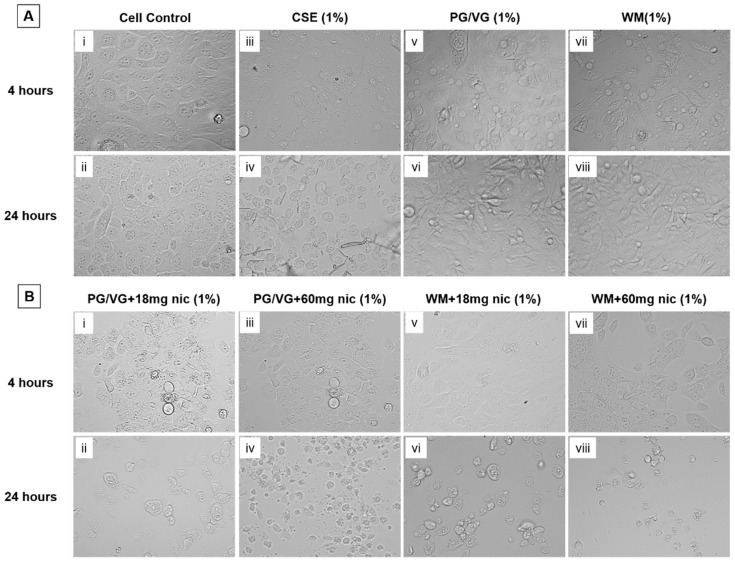
Morphological changes to BEAS-2B cells following electronic cigarette aerosol condensate and cigarette smoke exposure. (**A**) Cell controls, CSE and condensates without nicotine for 4 and 24 h treatments; (**B**) condensates with nicotine for 4 and 24 h treeatments. Brightfield microscopy images were taken at 40× objective of BEAS-2B cells following exposure to electronic cigarette condensates (PG/VG or WM) ± nicotine or CSE for 4 or 24 h and assessed for morphological changes in comparison with cell controls.

Viability and cellular membrane disruption data were supported by notable morphological changes to BEAS-2B cells following e-cigarette condensate exposure (1%). Control cells displayed patterns of typical growth and maintained structural integrity for both time points (Figure 7(Ai,Aii)), while cells exposed to CSE showed increasing loss of structural integrity (Figure 7(Aiii,Aiv)). With 4 h condensate treatments, the cell membrane is visibly intact (Figure 7(Av,Avii,Bi,Biii,Bv,Bvii)), but with 24 h treatment, significant cellular stress is noted with visible disruption of the cellular membrane (Figure 7(Avi,Aviii,Bii,Biv,Bvi,Bviii)). Higher nicotine concentration (60 mg) condensate exposures were observed to be necrotic (Figure 7(Biv,Bviii)).

### 3.4. Exposure to CSE Increases ACE2 Gene and Protein Expression in BEAS-2B Cells

**Figure 8:**
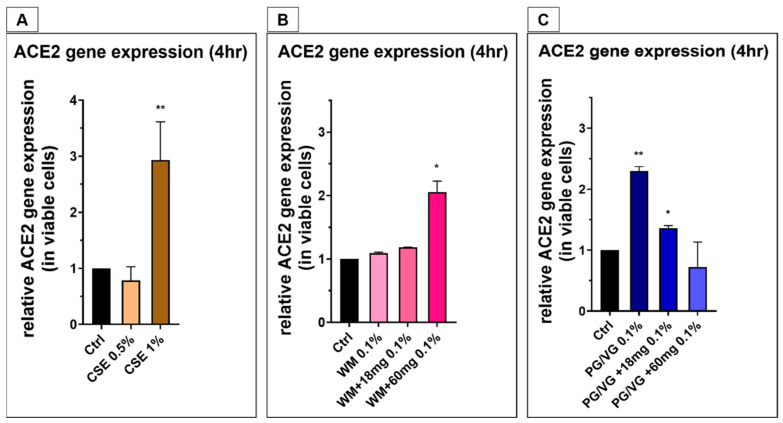
Angiotensin-converting enzyme-2 (ACE2) mRNA expression in BEAS-2B cells following cigarette smoke exposure and electronic cigarette condensates: BEAS-2B cells were exposed to (**A**) cigarette smoke extract (0.5% and 1%) or 0.1% concentration of electronic cigarette condensate generated from (**B**) watermelon-flavored e-liquid (Juicius Maximus) or (**C**) PG/VG ± nicotine for 4 h. Gene expression for ACE2 was assessed in treated cells and is presented as relative gene expression over cell controls, normalized with cytotoxicity for each treatment. Data are displayed as a median and interquartile range of *n* = 3–4 independent experiments. Nonparametric Mann–Whitney test was performed. ** *p* < 0.005, * *p* < 0.02.

**Figure 9:**
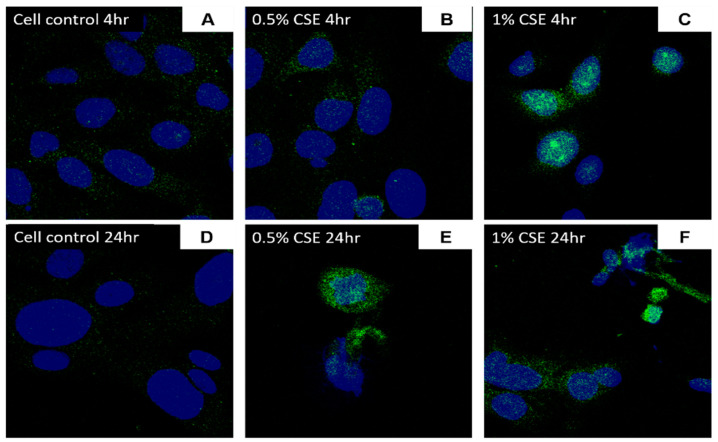
ACE2 protein expression in BEAS-2B cells following cigarette smoke exposure: BEAS-2B cells were exposed to cigarette smoke extract (CSE, 0.5% and 1%) for 4 or 24 h. Protein expression of ACE2 was assessed through immunofluorescence staining for 4 h (**B**,**C**) and 24 h (**E**,**F**) treatments in comparison with cell controls (**A**,**D**). Images at 40×.

Following 4 h of CSE (1%) treatment, a 3-fold ACE2 mRNA expression was observed in comparison to cell control (Figure 8A; *p* < 0.005). Immunofluorescence staining also revealed an overall increase in ACE2 protein expression with CSE treatment in comparison with untreated controls for 4 or 24 h treatment (Figure 9A–F). SAECs treated with 1% CSE also had a significant increase in ACE2 following 4 h exposure (Figure 12B). 4 h exposure to 0.5% and 1% CSE resulted in the observed localization of ACE2 expression towards the nuclei (Figure 9B,C), which is of similar localization and staining pattern as experiments with electronic cigarette condensates.

### 3.5. ACE2 Gene and Protein Expression Are Increased in BEAS-2B Cells Following Electronic Cigarette Aerosol Condensate Exposure

**Figure 10:**
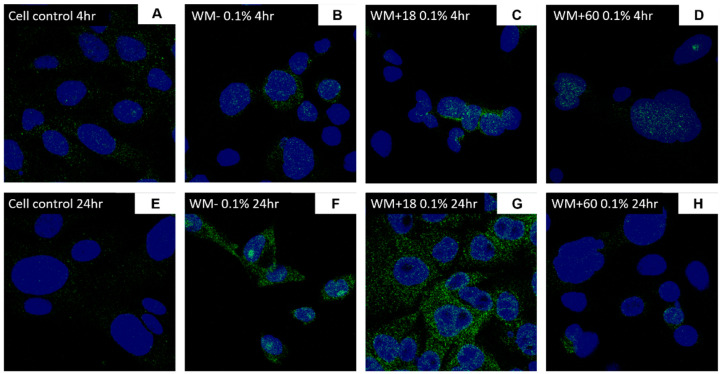
ACE2 protein expression of BEAS-2B cells following electronic cigarette aerosol (WM) exposure: BEAS-2B cells were exposed to a concentration (0.1%) of electronic cigarette condensate generated from watermelon e-liquid (WM) ± nicotine for 4 or 24 h. Protein expression of ACE2 was assessed through immunofluorescence staining for 4 h (**B**–**D**) and 24 h (**F**–**H**) treatments in comparison with cell controls (**A**,**E**). Images at 40×.

**Figure 11:**
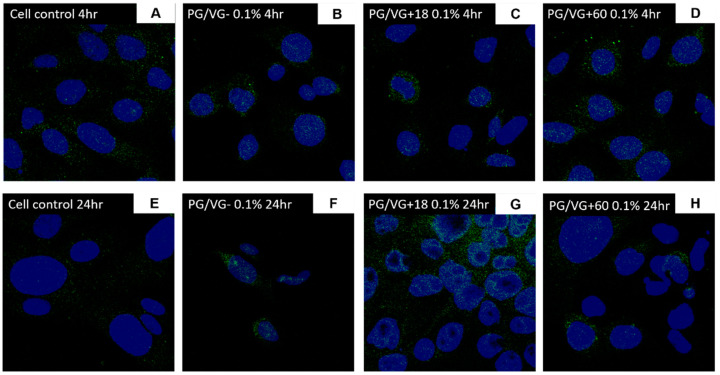
ACE2 protein expression of BEAS-2B cells following electronic cigarette aerosol (PG/VG) exposure: BEAS-2B cells were exposed to a concentration (0.1%) of electronic cigarette condensate generated from PG/VG ± nicotine for 4 or 24 h. Protein expression of ACE2 was assessed through immunofluorescence staining for 4 h (**B**–**D**) and 24 h (**F**–**H**) treatments in comparison with cell controls (**A**,**E**). Images at 40×.

**Figure 12:**
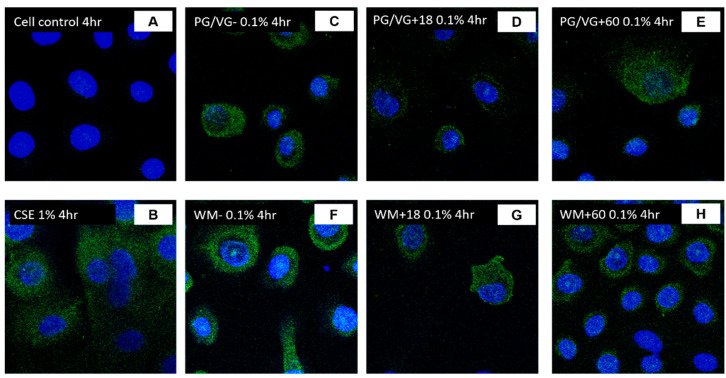
ACE2 protein expression of SAECs following CSE and electronic cigarette aerosol (PG/VG or WM) exposure: Small airway epithelial cells (SAECs) were exposed to a concentration (0.1%) of electronic cigarette condensate generated from PG/VG or WM ± nicotine or to 1% CSE for 4 h. Protein expression of ACE2 was assessed through immunofluorescence staining for 4 h (**B**–**H**) treatment in comparison with cell control (**A**). Images at 40×.

We proceeded with the utilization of a lower condensate concentration to measure the expression of ACE2 in BEAS-2B cells and SAECs. A two-fold increase in ACE2 mRNA expression was measured in cells treated with 0.1% WM+60 mg condensate (Figure 8B; *p* < 0.02). Increased ACE2 expression levels were also observed in cells treated with 0.1% PG/VG and PG/VG + 18 mg (Figure 8C; *p* < 0.005, *p* < 0.02), which may be accentuated due to lower viability from PG/VG condensate treatments. Immunofluorescence imaging revealed an increase in the expression of ACE2 on BEAS-2B cells with 4 h and 24 h posttreatment with condensates predominantly from nicotine-containing e-liquids. Untreated controls indicated that BEAS-2B cells had a degree of baseline ACE2 expression with diffuse staining pattern (Figure 9A,D; Figure 10A,E; Figure 11A,E). Treatment with 0.1% WM ± nicotine condensates for 4 h resulted in a slight increase in ACE2 expression compared with cell control (Figure 10B–D). ACE2 expression appears to be predominantly localized towards the nucleus in 0.1% WM+60 mg treated cells (Figure 10D). A similar trend was observed in PG/VG ± nicotine condensate 4 h exposure with a slightly more diffuse staining pattern in the cytoplasm (Figure 11B–D). The highest ACE2 expression was observed in cells treated with 0.1% WM− (Figure 10F) and 0.1% WM+ 18 mg (Figure 10G) condensates for 24 h. 24 h exposure to 0.1% WM+ 60 mg condensates resulted in a slight increase of ACE2 expression, albeit lesser than treatment with 18 mg/mL nicotine-containing condensates with localization towards the nucleus (Figure 10H). To a more moderate extent, the same trend was observed in PG/VG condensates (Figure 11F–H). SECs treated with both PG/VG and WM ± nicotine experienced an increase in ACE2 protein expression for all treatment groups (Figure 12). Immunostaining in SAECs appeared diffused, cytoplasmic with more membrane localization than seen in BEAS-2B.

## 4. Discussion

In this study, we tested a locally bought e-liquid (Juicius Maximus) alongside PG/VG alone and CSE. The WM e-liquid (0.1% WM + 60 mg/mL nicotine) and PG/VG (0.1% PG/VG and 0.1 PG/VG + 18 mg/mL nicotine PG/VG) significantly amplified ACE2 gene expression when mixed with a high concentration of nicotine along with 1% CSE. Further, immunofluorescence imaging displayed an increase in ACE2 protein expression in BEAS-2Bs following 4 and 24 h, and SAECs following 4 h, post-exposure to PG/VG and WM (±added nicotine) condensates, with the strongest expression observed with condensates containing 18 mg/mL of nicotine for BEAS-2Bs. We are not able to justify the exact reason for the disparity between gene and protein expression data in BEAS-2Bs. We, however, believe that a nicotine concentration could be having an effect, along with early mRNA expression. All treatments with e-cigarette condensates resulted in an increase in ACE2 protein expression in SAECs. Here we have shown that ACE2 is upregulated in both bronchial epithelial cells along with small airway epithelial cells. This is the first study using electronic cigarette condensates in investigating viability/cytotoxicity and ACE2 expression. This methodology is novel to our research group.

Our BEAS-2B cytotoxicity data shows that at 4 h of exposure, 0.1% WM+ 60 mg did not result in a reduction in viability nor a significant release of LDH. In contrast, higher concentrations of condensate generated from nicotine-containing mixtures were indeed significantly cytotoxic, as were all concentrations of nicotine-free condensates for both 4 and 24 h exposures. Ji et al. have previously shown that upon the separation of CSE into a nicotine-free section and nicotine-containing section, more significant cytotoxicity was observed in BEAS-2B cells induced by the nicotine-free section of CSE than the nicotine-containing section of CSE [32]. This appears to also occur with our findings as treatment with nicotine-free condensates resulted in significantly more reduction in viability than treatment with nicotine-containing condensates. We do, however, see a significant reduction in viability in condensate treatment with 60 mg/mL nicotine content.

LDH release was measured to be significantly increased in cells following 24 h of exposure to concentrations (1% and 0.5%) of condensate generated from mixtures containing nicotine (18 and 60 mg/mL). Comparative to viability data, we see here that PG/VG or WM with nicotine were driving cellular disruption and a reduction in cell membrane integrity. Brightfield images of treated cells also clearly show that at 4 h, membrane disruption is yet to occur, while 24 h images show that lysis is occurring in cells and membrane integrity is significantly reduced. The novelty here lies in our use of electronic cigarette condensates to obtain our toxicological results and our preliminary results in primary small airway epithelial cells. E-cigarette use, particularly with higher nicotine concentration, is an avoidable risk factor with potentially deadly implications concerning the contraction of SARS-CoV-2.

The deadly spread of SARS-CoV-2 and the development of COVID-19 is influenced by numerous and broad factors [33]. Increased virus susceptibility and COVID-19 demographic representation have been identified for tobacco smokers. Now we provide novel and preliminary results to the growing evidence that electronic cigarette vaping is detrimental and may increase susceptibility to SARS-CoV-2 infection. The large airways are an early interaction point for the virus before the development of the disease, and here, we have used stimulants in e-cigarette condensates and CSE to focus on the role of virus interaction with large airway epithelial cells along with primary SAECs.

The capacity for modern electronic cigarette devices to deliver large doses of nicotine is of great concern; thus, we have used both 18 mg/mL and 60 mg/mL and made a comparative assessment. Our results show that condensates generated from PG/VG and-flavored e-liquids, particularly with a nicotine content of 18 mg/mL, can increase the expression of ACE2. An increase in ACE2 expression was observed alongside distinct cellular distress seen with both short and prolonged exposures. This indicates that vaping may facilitate an increased vulnerability to viral infection in the lung epithelial cells of e-cigarette users.

Previously, programmed cell death has been shown in BEAS-2B cells and macrophages following 24 h exposure to unflavored ENDS aerosol (PG/VG ± nicotine) [34]. In our study, treatment with PG/VG ± nicotine causes a reduction in viability, and PG/VG with nicotine causes cell membrane disruption and an increase in LDH release. Electronic cigarette exposure has also been shown to increase DNA damage and induce cell death in a human epithelial cell line regardless of nicotine concentration [35]. Combined with the detection of carcinogens in e-cigarette end products [36], DNA damage could indicate the potential progression to tumorigenesis. Along with our data, the complexity of the risk for acute and chronic respiratory insult is fueled by the interplay of substances in e-liquids that can disrupt cells and lead to dysfunctional airways. An awry concoction with a misjudged and extreme concentration of nicotine could prime the lung for viral infection or severe acute respiratory distress.

Prior to the pandemic of 2020, the US was inundated in 2019 with hospital admissions from young, healthy individuals who had succumbed to severe lung disease driven by vaping (EVALI) [37]. Mice exposed to aerosols from counterfeit cartridges (linked to the EVALI epidemic) were recently shown not to affect ACE2, furin, or TMPRSS protein expression [38]. However, sub-chronic exposure to e-cigarette aerosol (PG + nicotine) in female mice triggered an inflammatory response and an increase in ACE2 protein expression, mediated by the α7 nAChR receptor [39]. Our results strengthen this finding from Wang et al. in that vaping can result in an increase in ACE2.

The addition of vitamin E acetate to e-liquids is linked to the 2019 outbreak of EVALI and is a colossal black flag for the potential for electronic cigarette vaping to become an enormous burden on the health of global populations. It is now pivotal to expand experiments to include testing for a larger sample of e-liquid combinations. The modifiable nature of these new generation devices is cause for alarm.

With rigorous and robust e-cigarette research emerging, the scientific and clinical health care community are confidently grasping the genuine safety concerns of vaping and fear the long-term effects yet to materialize clinically. Sadly, in such global trying times, we witness the exploitation of the pandemic by the manufacturers and marketing of e-cigarettes and heat-not-burn devices [40]. Heat-not-burn devices are another modern nicotine delivery system that heats tobacco plugs to high-temperature, producing smoke to inhale without fully igniting the tobacco. Therefore, a new generation of addicted vapers is growing alongside global users of heat-not-burn devices, and so is the risk of short- and long-term vape-driven disease [41,42]. A stronger stance on the safety and necessity of e-cigarettes should be held to reduce the risk of virus spread and avoid future outbreaks of EVALI. We are on the right path towards recognizing the harm in e-cigarettes [43].

The limitations of our preliminary results are mostly linked to the vast possibilities lying in the number of variables that come with the act of vaping. Our study also only utilized one design or make of e-cigarette, which was set to one power output for consistency. Here we have not measured the inflammatory profile following stimulation and have limited this study to a bronchial epithelial cell line with some data shown from primary small airway epithelial cells, but we plan to expand on this in our follow-up studies. Our interest also lies in the expression profiles of type 2 pneumocytes. Quantification of ACE2 expression and other interacting proteins (furin, TMPRSS2) could be further explored in human tissue and murine models.

## 5. Conclusions

We provide here evidence that, along with tobacco smoking, the use of electronic cigarettes is not safe and can increase the expression of ACE2 in the lung. We reiterate the strong caution to be taken in the promotion of electronic cigarettes, particularly in the midst of this pandemic. Developing and matured human lungs are not designed for anything but the inhalation of natural air. Vaping is an unsafe practice, and along with cigarette smoking, may lead to increased susceptibility to viral infection and the progression to severe respiratory distress or disease.

## Data Availability

All the data generated and analyzed during the current study are available on reasonable request to the corresponding author.
